# Does Cigarette Smoking Increase Traffic Accident Death During 20 Years Follow-up in Japan? The Ibaraki Prefectural Health Study

**DOI:** 10.2188/jea.JE20170330

**Published:** 2019-05-05

**Authors:** Ayaka Igarashi, Jun Aida, Toshimi Sairenchi, Toru Tsuboya, Kemmyo Sugiyama, Shihoko Koyama, Yusuke Matsuyama, Yukihiro Sato, Ken Osaka, Hitoshi Ota

**Affiliations:** 1 Department of International and Community Oral Health, Tohoku University Graduate School of Dentistry, Sendai, Japan; 2 Health Prevention Division, Health and Welfare Department, Ibaraki Prefecture Office, Mito, Japan; 3 Department of Public Health, Dokkyo Medical University School of Medicine, Tochigi, Japan; 4 Ibaraki Health Plaza, Mito, Japan; 5 Ibaraki Health Service Association, Mito, Japan; 6 Department of Community Medical Supports, Tohoku Medical Megabank Organization, Tohoku University, Sendai, Japan; 7 Department of Global Health Promotion, Graduate School of Medical and Dental Sciences, Tokyo Medical and Dental University (TMDU), Tokyo, Japan; 8 Japan Society for the Promotion of Science, Tokyo, Japan; 9 Department of Quality and Safety of Oral Healthcare, Radboud University Medical Center, Nijmegen, Netherlands

**Keywords:** cigarette, smoking, traffic accident, cohort study

## Abstract

**Background:**

Annually, more than 1.2 million deaths due to road traffic accidents occur worldwide. Although previous studies have examined the association between cigarette smoking and injury death, the mortality outcome often included non-traffic accident-related deaths. This study aimed to examine the association between cigarette smoking and traffic accident death.

**Methods:**

We conducted a prospective cohort study using data from the Ibaraki Prefectural Health Study conducted from 1993 through 2013. The cohort included 97,078 adults (33,138 men and 63,940 women) living in Ibaraki Prefecture who were aged 40–79 years at an annual health checkup in 1993. We divided participants into four smoking status groups: non-smokers, ex-smokers, and current smokers who smoked <20 and ≥20 cigarettes per day. Hazard ratios (HRs) of traffic accident death were calculated using a Cox proportional hazards model.

**Results:**

During 20 years of follow-up, the average person-years of follow-up were 16.8 and 18.2 in men and women, respectively. Among men, after adjusting for age and alcohol intake, HRs for traffic accident death among current smokers of <20 cigarettes/day and ≥20 cigarettes/day compared to non-smokers were 1.32 (95% confidence interval [CI], 0.79–2.20) and 1.54 (95% CI, 0.99–2.39), respectively. In contrast, among women, we found no association between smoking status and traffic accident deaths.

**Conclusion:**

In this prospective cohort study, we found a positive association, though marginally significant, between smoking and traffic accident death among men in Japan. Among women, because of the smaller number of deaths among smokers, adequate estimation could not be obtained.

## INTRODUCTION

Every year, more than 1.2 million people die and 50 million people are injured due to road traffic accidents worldwide.^1^ Road traffic accidents are the main cause of death among young people aged 15–29 years.^1^ Traffic accident injuries also deteriorate quality of life and disability-adjusted life years.^2^^,^^3^ Economic loss caused by road traffic accidents is also quite high, accounting for about 3% of the total gross domestic product of all countries worldwide.^1^ Although many road traffic policies have been implemented to prevent traffic accidents, there have, nevertheless, been a lot of traffic accidents in the world.^2^ The Sustainable Development Goals adopted at the United Nations Summit in 2015 included two targets on road safety.^4^^–^^6^ These goals are to halve the number of global deaths and injuries from road traffic accidents by 2020 and to provide access to safe, affordable, accessible, and sustainable transport systems for all people, with special attention to the needs of those in vulnerable situations, by expanding public transport by 2030.^4^^–^^6^ Prevention of road traffic accidents is an important public health issue.

Cigarette smoking is reported to be a risk factor for traffic accident deaths.^7^^,^^8^ Previous studies have reported that smokers have a higher risk of injury death (ie, death due to injury), including traffic accident death, than do non-smokers; this is because smoking physically ignites fires, smokers tend to have more underlying diseases that affect accident rates, and smokers are more likely to engage in risky behaivors.^8^ In addition, smoking is considered to increase the risk of car driver’s accidents because smoking during driving distracts the driver, impairing their attention.^9^^–^^11^ Although previous studies have examined the association between cigarette smoking and injury deaths, the mortality outcome often included non-traffic accident deaths.^7^^,^^8^^,^^12^ In addition, only one ecological study has been conducted to specifically examine the association between smoking and traffic accident death in Japan.^13^ To establish more concrete evidence regarding this association, we aimed to examine the association between smoking and traffic accident deaths, using a prospective cohort in Japan.

## MATERIAL AND METHODS

### Study cohort and population

**Figure 1.**
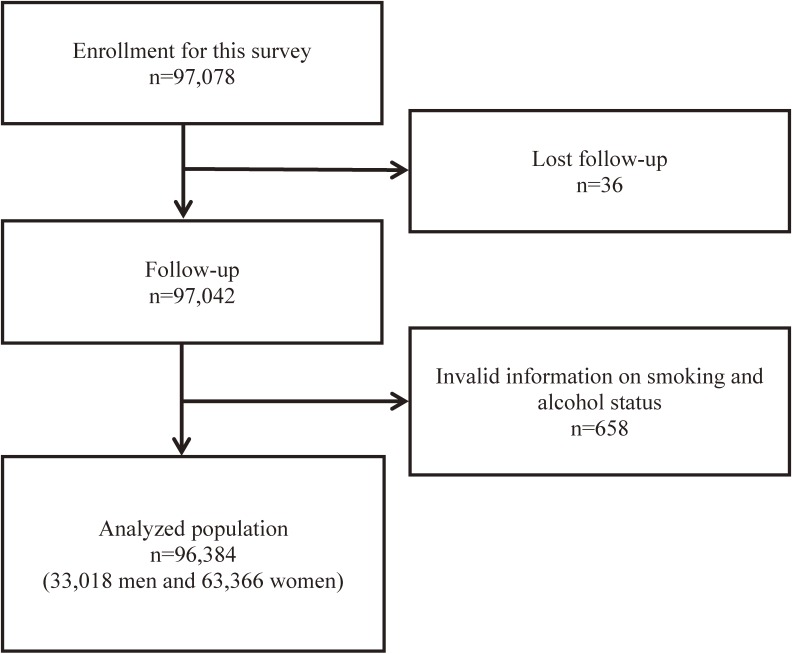
Participant flow diagram from the Ibaraki Prefectural Health Study

We used data collected in the Ibaraki Prefectural Health Study, a prospective cohort study.^14^ Briefly, the cohort included 97,078 adults (33,138 men and 63,940 women) living in Ibaraki Prefecture who were aged 40–79 years when they participated in annual community-based health checkups in 1993. This cohort was followed up until the end of 2013. Thirty-six individuals could not be followed up (0.7%). The follow-up data were censored when participants moved to another municipality from the municipality where they lived at the baseline. A total of 658 individuals were excluded from the current analysis due to lack of information on smoking and alcohol habits. Finally, 96,384 individuals (33,018 men and 63,366 women) were included in the analysis (Figure 1). This study was approved by the Ethics Committee of Ibaraki Prefecture.

### Outcome measurement

Death occurring during the 20-year follow-up period was used as our outcome variable. Death and emigration dates were obtained from local government records. Dates and causes for all deaths recorded in the cohort were obtained, except for those among individuals who had moved to other municipalities. The International Classification of Diseases (ICD), 9^th^ (1993–1994) and 10^th^ (1995–2012) revisions were applied to code the causes of death. Traffic accident deaths were identified as code V01–99 in ICD-9 and ICD-10. These traffic accidents included accidents caused not only by cars, but also by bicycle, bike, trains, planes, and other vehicles.

### Independent variable

Data on smoking status, number of cigarettes smoked per day, and alcohol intake (obtained in “go”, which is a Japanese traditional unit [1 go = 180 mL]) were obtained from health checkups listed in local government records at baseline.

Participants were divided into four smoking status categories: non-smokers, ex-smokers, current smokers who smoked <20 cigarettes per day, and current smokers who smoked ≥20 cigarettes per day. Alcohol intake was divided into four categories: teetotaler (abstains from alcohol), sometimes consumes alcohol, consumes <3 go of alcohol every day, and consumes ≥3 go of alcohol every day.

### Statistical analysis

We used Cox proportional hazards regression models stratified by sex to calculate hazard ratios (HRs) and 95% confidence intervals (CIs) for traffic accident death. Age and alcohol intake were included as covariates. Significance level was set at *P* < 0.05 (two-sided). Continues variables are presented as mean (standard deviation [SD]). All statistical analyses were conducted using SAS version 9.1.3 (SAS institute, Inc., Cary, NC, USA).

## RESULTS

Mean ages of the 33,018 men and 63,366 women at baseline (1993 checkup) were 60.7 (SD, 10.0) and 58.1 (SD, 10.3) years, respectively. During the 20-year follow-up period (1993–2013), mean person-years of follow-up were 16.8 and 18.2 person-years in men and women, respectively. There were 168 (0.5%) and 128 (0.2%) traffic accident deaths among men and women, respectively.

Table 1 shows baseline characteristics stratified by smoking status and sex. Non-smokers showed a lower rate of traffic accident deaths: event rates per 1,000 person-years in men were 0.24, 0.30, 0.36, and 0.32 among non-smokers, ex-smokers, and current smokers of <20 cigarettes/day and ≥20 cigarettes/day, respectively. In contrast, among women, the rate of traffic accident deaths was not lower among non-smokers because the numbers of smokers and death events were smaller. Among women, 94.4% were non-smokers, and the numbers of traffic accident deaths were 1, 0, and 0 among ex-smokers and current smokers of <20 cigarettes/day and ≥20 cigarettes/day, respectively. In relation to alcohol intake, compared with non-smokers and ex-smokers, current smokers (<20 and ≥20 cigarettes/day) of both sexes consumed more alcohol.

**Table 1.** Baseline characteristics by smoking status: the Ibaraki Prefectural Health Study

		Men				Women			
	
		Non-smokers	Ex-smokers	Current smokers		Non-smokers	Ex-smokers	Current smokers	
	
				<20 cigarettes/day	≥20 cigarettes/day			<20 cigarettes/day	≥20 cigarettes/day
Number of individuals		7,335	9,155	5,125	11,403	59,832	461	2,021	1,052
Person-years		127,605	152,646	79,956	193,438	1,091,277	7,807	34,492	17,926
Number of events		31	46	29	62	127	1	0	0
Rate of events (per 1,000 person-years)		0.24	0.30	0.36	0.32	0.12	0.13	0	0
Age, years, mean (SD)		60.8 (10.2)	62.6 (9.5)	63.5 (9.1)	57.8 (10.0)	58.3 (10.2)	56.4 (11.5)	56.3 (11.2)	53.5 (9.7)
Alcohol intake, %									
Teetotaler		43.6	35.3	32.6	30.6	91.9	65.1	69.3	66.3
Sometimes		16.0	14.1	13.2	11.7	5.5	19.1	15.3	12.6
Every day	<3 units/day	37.2	45.6	49.1	45.9	2.6	14.8	14.8	17.4
	≥3 units/day	3.1	4.9	5.1	11.8	0.0	1.1	0.5	3.7

SD, standard deviation.

Table 2 shows HRs and 95% CIs for traffic accident death according to smoking status. Among men, compared to non-smokers, the multivariate HRs for traffic accident death were greater among current smokers. Among men, after adjustment for age and alcohol intake, compared to non-smokers, HRs in current smokers of <20 cigarettes/day and ≥20 cigarettes/day were 1.32 (95% CI, 0.79–2.20) and 1.54 (95% CI, 0.99–2.39), respectively. When the interaction between smoking and alcohol consumption for traffic accident deaths was examined, no significant interaction between alcohol consumption and smoking was observed. In contrast, among women, we found no association between smoking status and traffic accident deaths.

**Table 2.** Hazard ratios of traffic accident death according to participant smoking status

		Rate of events (per 1,000 person-years)	Age-adjusted			Fully-adjusted^a^		
	
			Hazard ratio	95% CI	*P-value*	Hazard ratio	95% CI	*P-value*
Men								
Non-smokers		0.24	reference			reference		
Ex-smokers		0.30	1.14	0.72, 1.79	*0.58*	1.13	0.72, 1.79	*0.59*
Current smokers	<20 cigarettes/day	0.36	1.32	0.80, 2.19	*0.28*	1.32	0.79, 2.20	*0.28*
	≥20 cigarettes/day	0.32	1.60	1.03, 2.47	*0.04*	1.54	0.99, 2.39	*0.06*
Women								
Non-smokers		0.12	reference			reference		
Ex-smokers		0.13	1.19	0.17, 8.53	*0.86*	1.21	0.17, 8.73	*0.85*
Current smokers	<20 cigarettes/day	0	—	—	—	—	—	—
	≥20 cigarettes/day	0	—	—	—	—	—	—

CI, confidence interval; —, no observation.

^a^In the fully-adjusted model, we adjusted for age and alcohol intake.

## DISCUSSION

In this prospective cohort study, among men, current smokers of ≥20 cigarettes/day had marginally significantly higher risk of traffic accident death compared to non-smokers. In contrast, among women, because of the low number of traffic accident deaths, no association was observed.

The presence of an association among men is consistent with the findings of previous studies. A previous prospective cohort study reported a significant dose-response association between number of cigarettes smoked and unintentional injury deaths, including traffic accident deaths, independent of age, race, gender, alcohol intake, seatbelt use, education, and marital status.^7^ In addition, a review reported that the risk of motor vehicle crashes was two times higher in smokers compared with non-smokers, after stratifying by age, driving experience, education, and alcohol intake.^12^

There is a possible mechanism that might explain the association between smoking and traffic accident deaths. Distraction or impaired attention due to smoking during driving a car may have contributed to the rate of accidents observed. A series of smoking behaviors, such as searching for cigarettes, lighting a cigarette, dropping ash on an ashtray, and extinguishing a burning cigarette are considered to distract the driver.

Contrary to the significant association found among men, we found no significant association between smoking status and traffic accident death among women. A recent meta-analysis of cohort studies supports our null result; the meta-analysis found no significant association between smoking and injury death among women.^8^ We could not calculate HRs of traffic accident death among women because the number of smokers was small and an event did not occur in this group. Therefore, a marginally significant association was observed only among men in this study. In our study, a larger sample of women would have been required to obtain a more robust estimation.

We believe that the present study has important public health implications. Risks associated with smoking during driving should be announced extensively in public. In addition, prohibition of smoking during driving by law should be considered because the use of mobile phones during driving is currently forbidden by law in many countries.^15^ In some countries, smoking in cars is forbidden by law to prevent secondhand smoke.^16^^–^^18^ In addition to preventing secondhand smoke, reducing driving accidents should be included as a reason for prohibiting smoking in cars.

This study had certain limitations. First, information on the smoking habits of participants did not specifically assess smoking during driving. In addition, smoking habits at baseline may have changed during the follow-up period. However, the results of this study are robust, as the non-differential exposure misclassification would be expected to attenuate real effect estimates, leading to the loss of ability to detect true effects. Second, this study used death certificates from local government records to determine the number of deaths in the cohort. However, the situations surrounding the traffic accidents were not recorded. Therefore, the traffic accident deaths included not only car accidents, but also deaths due to other vehicular accidents, such as train and water traffic accidents, as well as people killed in traffic accidents caused by other drivers’ mistakes. Some of these accidents might not be related to smoking behavior. Therefore, the present study may have underestimated the association between smoking and traffic accident deaths. Third, previous studies suggested that people with lower socioeconomic status are more likely to have car accidents.^19^^,^^20^ Therefore, socioeconomic status may be a confounding factor in the association between smoking and traffic accident deaths. Our dataset did not include any variables with which we could assess socioeconomic status. Future studies should therefore consider this variable when examining the association between smoking during driving and traffic accident death.

The primary strengths of our study were that the follow-up period was 20 years, which is considerably longer than those of previous studies. In addition, we used a highly reliable death registry and had extremely low loss to follow-up (0.7%). The high follow-up rate reduces possibility of selection bias.

### Conclusion

In this prospective cohort study, we found a positive association, though marginally significant, between smoking and traffic accident death among men in Japan. Among women, because of the smaller number of death among smokers, adequate estimation could be obtained.
